# Security, Violent Events, and Anticipated Surge Capabilities of Emergency Departments in Washington State

**DOI:** 10.5811/westjem.2016.10.30271

**Published:** 2017-03-03

**Authors:** Jonathan S. Weyand, Emily Junck, Christopher S. Kang, Jason D. Heiner

**Affiliations:** * 212th Combat Support Hospital, Rhine Ordnance Barracks, Germany; † Landstuhl Regional Medical Center, Department of Emergency Medicine, Landstuhl, Germany; ‡ University of Washington, Division of Emergency Medicine, Seattle, Washington; § Madigan Army Medical Center, Department of Emergency Medicine, Tacoma, Washington; ¶ PeaceHealth Peace Island Medical Center, Department of Emergency Medicine, Friday Harbor, Washington

## Abstract

**Introduction:**

Over the past 15 years, violent threats and acts against hospital patients, staff, and providers have increased and escalated. The leading area for violence is the emergency department (ED) given its 24/7 operations, role in patient care, admissions gateway, and center for influxes during acute surge events. This investigation had three objectives: to assess the current security of Washington State EDs; to estimate the prevalence of and response to threats and violence in Washington State EDs; and to appraise the Washington State ED security capability to respond to acute influxes of patients, bystanders, and media during acute surge events.

**Methods:**

A voluntary, blinded, 28-question Web-based survey developed by emergency physicians was electronically delivered to all 87 Washington State ED directors in January 2013. We evaluated responses by descriptive statistical analyses.

**Results:**

Analyses occurred after 90% (78/87) of ED directors responded. Annual censuses of the EDs ranged from < 20,000 to 100,000 patients and represented the entire spectrum of practice environments, including critical access hospitals and a regional quaternary referral medical center. Thirty-four of 75 (45%) reported the current level of security was inadequate, based on the general consensus of their ED staff. Nearly two-thirds (63%) of EDs had 24-hour security personnel coverage, while 28% reported no assigned security personnel. Security personnel training was provided by 45% of hospitals or healthcare systems. Sixty-nine of 78 (88%) respondents witnessed or heard about violent threats or acts occurring in their ED. Of these, 93% were directed towards nursing staff, 90% towards physicians, 74% towards security personnel, and 51% towards administrative personnel. Nearly half (48%) noted incidents directed towards another patient, and 50% towards a patient’s family or friend. These events were variably reported to the hospital administration. After an acute surge event, 35% believed the initial additional security response would not be adequate, with 26% reporting no additional security would be available within 15 minutes.

**Conclusion:**

Our study reveals the variability of ED security staffing and a heterogeneity of capabilities throughout Washington State. These deficiencies and vulnerabilities highlight the need for other EDs and regional emergency preparedness planners to conduct their own readiness assessments.

## INTRODUCTION

### Background and Importance

Over the past 15 years, violent threats and acts against hospital patients, staff, and providers have increased and escalated.^1^ The non-fatal assault rate of healthcare workers has been reported to be up to four times the rate for all private–sector industries.^2^ Hospital-based shootings nearly doubled from 2000–2011.^3^ Within the hospital, the leading area for violence is the emergency department (ED) given its 24/7 operations in patient care and as the admissions gateway,^4^ with assault rates as high as 1.1 per 100,000 ED employee hours per year.^5^ A study of EDs in Cincinnati reported 98% of nurses and 96% of physicians had been verbally abused, and 67% of nurses and 51% of physicians had been physically abused while at work.^6^ Another study in Michigan found the average ED healthcare worker was physically threatened four times per year and assaulted at least once per year.^7^

Concurrently, EDs are often the center for influxes of patients, crowds, media, and traffic during mass casualty events that include natural disasters and terrorist attacks. Violent incidents, such as in 2009 at Fort Hood, 2012 in Aurora, CO, in New York City during Hurricane Sandy, and during the 2013 Boston Marathon, accentuate the importance of ED and hospital campus-specific plans to rapidly augment hospital security and operations. Although accredited hospitals are required to have an emergency management plan, because of costs and a lack of standardization, EDs and hospitals employ a variety of security protocols ranging from in-house “rapid response teams” to reliance on local law enforcement.^8^ No standardized requirements or recommendations for emergency planning exist, and anecdotal lack of familiarity with ED and hospital security plans further complicate ED personnel safety and operations, which may be magnified during an acute surge or mass casualty event.

### Goals of This Investigation

Growing research in ED violence exists, yet there is an absence of detailed statewide or comprehensive characterization of ED security and resources for normal operations and mass casualty event response. This investigation had three objectives: to assess the current security of Washington State EDs; to estimate the prevalence of and response to day-to-day threats and violence in Washington State EDs; and to appraise the Washington State ED security capability to respond to a mass casualty event.

## MATERIALS AND METHODS

### Study Design

A voluntary, blinded, 28-question Web-based survey developed by emergency physicians was electronically delivered to all 87 Washington State ED directors in January 2013 (Appendix). Two senior physician-authors, with disaster medicine focus and publications, created the survey based on observations and reports of numerous hospital emergency management plans, which was to serve as an initial assessment of a potentially overlooked topic. As a result, the survey did undergo a formal validation phase. Review and approval by the Washington State American College of Emergency Physicians (ACEP) and local institutional review board were obtained. Multiple-choice questions pertained to basic ED demographics, current security protocols and resources, estimated prevalence and types of threats and violent incidents, and ability of security to respond to acute events (Table 1). Four subsequent monthly email participation reminders were sent and final responses collected in June 2013.

**Population Health Research Capsule**

What do we already know about this issue?

Over the past 15 years, violence within hospitals and the frequency of mass casualty events has increased and escalated. The emergency department is the leading area for this violence and influx.

What was the research question?

Within Washington State EDs, what are the frequencies of violent events, basic response protocols, and surge capabilities?

What was the major finding of the study?

Our study reveals the variability of ED security staffing and a heterogeneity of capabilities throughout WA State.

How does this improve population health?

This initial and unprecedented survey highlights the need for other EDs and regional emergency preparedness planners to conduct their own readiness assessments and examine their protocols.

### Data Collection and Processing

ED directors were aware that their responses would remain anonymous via the Web-based survey collection tool. Missing survey item answers were treated as no responses. We evaluated data using univariate descriptive statistical analyses. The data were based solely on the responses of the ED directors and were not compared to police or hospital reports.

## RESULTS

### Demographics

Seventy-eight of 87 (90%) Washington State ED directors responded between January and June 2013 (Table 1). A majority reported one (45%) or two (19%) security personnel on duty. Twenty-one (28%) responded zero or “not applicable.” Nearly two-thirds (63%) had 24-hour security coverage. Security personnel were provided to 60% of EDs by the hospital, 17% by private companies, and 9% by local law enforcement agencies. Security personnel training was provided by 45% of hospitals or healthcare systems, while 11% used an agency or contractor-sponsored course, 15% relied on prior training, and 3% used a non-employer sponsored training course. Three (4%) reported no prior or formal training for security personnel.

### Prevalence of and Response to Threats and Violence

**Figure 1:**
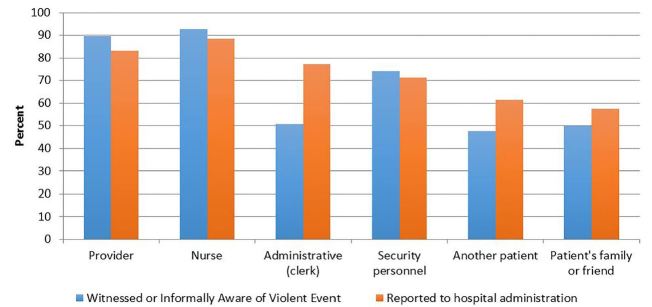
Percent distributions of violent threats or acts witnessed by or reported to emergency department (ED) directors and reported to hospital administration.

Sixty-nine of 78 (88%) ED directors witnessed or heard violent threats or acts occurring in their ED. Of these respondents, 93% had witnessed these threats/acts directed towards nursing staff, 90% towards physicians, 74% towards security personnel, and 51% towards administrative personnel. Nearly half (48%) noted incidents directed towards another patient, and 50% towards a patient’s family or friend (Figure 1). These events were variably reported, according to ED directors’ recollection, to the hospital administration—most often for incidents involving nurses (89%) and providers (83%). Incident reporting rates were lower for administrative staff (77%) and security personnel (71%), and lowest when directed towards another patient (62%) and or their family or friends (58%) (Figure 1).

Fifty-nine of 75 (79%) EDs had plans to notify and receive additional security personnel. Twenty-three (31%) would be able to receive additional security personnel from within the hospital in under five minutes, 35% within 5–15 minutes, and 8% within 16–30 minutes. Five (7%) EDs would have to wait for 30 or more minutes. Thirty-four of 75 (45%) EDs reported that the general consensus of their ED staff was that the current level of their security was inadequate.

### Response to an Acute Surge or Mass Casualty Event

**Figure 2:**
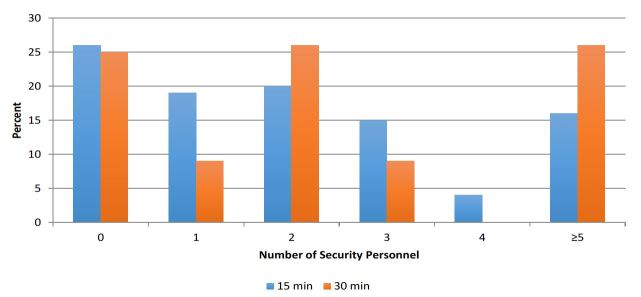
Number of security personnel that could respond within 15 and 30 minutes of activation of the hospital emergency management plan.

After an acute surge or mass casualty event, 18 of 69 (26%) respondents believed the availability and size of the initial additional security response would be adequate, while 35% did not, and 39% were unsure. The number of security personnel that could present within 15 and 30 minutes upon activation of their hospital’s emergency management plan varied (Figure 2), including 26% reporting no additional security would be available within 15 minutes and 25% reporting additional personnel within 30 minutes.

The ED security personnel source during normal operations and responding to an acute surge event varied. Additional security personnel would be provided by 24 of 61 (39%) hospitals, while 8% would receive support from a private or contracted company and 46% depended on local law enforcement. When asked about the highest level of assurance that additional security personnel would be available and respond to an acute surge or mass casualty event, 38% reported additional security was already present on the hospital campus, arranged through a formal contract, or coordinated via a memorandum of understanding or agreement. Twelve (20%) reported reliance on an unwritten agreement, and 41% did not know.

Thirty-nine of 61 (64%) ED directors reported that points of entry and egress from the hospital could be secured within 15 minutes (Table 2). When asked about specific scenario response effectiveness, 57% believed that their security would be able to handle a violent criminal or terrorist in the ED, and 59% and 56% felt security could handle a surge of patients and of patients’ family and friends, respectively, arriving within one hour. If an acute surge of patients greater than the current capacity of the ED and its waiting room occurred within an hour, 61% reported planned policies to limit access of visitors. Thirty-eight (62%) did not know of or have a security protocol to control traffic for incoming patients, additional hospital personnel, medical equipment suppliers, responding agencies, and media. Fifteen (25%) did not know of or have a security plan to enforce the quarantine of contaminated and contagious patients. Nineteen (31%) respondents did not have or know of a security protocol to secure contaminated items, high-value possessions, or firearms. Seventeen (28%) were unaware of securing and maintaining a chain of custody for potential forensic evidence (Figure 2).

## DISCUSSION

A 2011 ACEP policy statement advocated that hospitals have a responsibility to “provide a best-practices security system, including adequate security personnel, sufficient training of personnel, physical barriers, surveillance equipment, and other security components, coordinate … with local law enforcement agencies, [and] develop written ED protocols for violent situations occurring in the ED to ensure the safety of patients and health care workers alike.”^9^ Our study reveals variable ED security staffing and training and a heterogeneous collection of plans and capabilities throughout Washington State. Although disaster plans exist, a number of common potential deficiencies were apparent, such as uniformity of security training, reporting of violent acts, and specific protocols for securing firearms, hospital resources, and forensic evidence. Concerning vulnerabilities exist including lack of additional and readily-available security, capability to rapidly secure access to EDs, and crowd and traffic control.

### Demographics

While nearly two-thirds (63%) of EDs had 24-hour security personnel coverage, 28% reported no assigned security personnel. A 2012 study in New Jersey found that small-town hospitals in areas with low crime indices or violent crime rates implemented the fewest security features. Despite hypotheses that small EDs in low crime areas would need less security protection, these facilities had more violent acts than large hospitals in areas of low and high crime rates.^5^ In Washington State, we found that lower census EDs more often have no or only part-time security presence. We recommend full-time dedicated security presence for all EDs, or at least full-time hospital security that can quickly be activated to the ED and planned coordination with local law enforcement.

### Prevalence of and Response to Violent Threats and Acts

Congruent with past investigations, we found ED personnel are likely to witness or experience workplace violence. We also found that violence was common against other patients and their families and friends as well. This reinforces that improved security measures are needed not just to protect those who work in EDs, but the patients and other visitors who seek care and safety in EDs.

Another intriguing finding was the gap between violent threats and acts witnessed or heard about and the subsequent reporting rate to administration. Several previous studies have reported similar patterns, including a 2006 study that found only 26% of ED providers and 45% of ED employees in general filed formal reports after experiencing a spectrum of violent acts.^6^ Another study in 2011 found only 35% of violence by patients and 55% by visitors were reported.^10^ We recommend that ED supervisors support a culture of reporting and an easy method to file reports without punitive consequences. Having accurate data regarding the locations, times, and natures of these events will help hospitals and government systems to further secure the workplaces and healthcare settings for staff and patients alike and to identify problems and gain resources in this effort.

### Response to an Acute Surge or Mass Casualty Event

The increased national incidence of ED and hospital-based shootings reinforce the importance of hospital security, which may deter or rapidly respond to “active shooters” or other imminent threats. A 2008 study identified that, despite having a disaster plan and conducting disaster drills, one out of six Los Angeles County 911-receiving hospitals did not have a protocol for hospital lockdown or involved the local police department.^11^

Of concern in Washington State as well, we found nearly half (43%) of ED directors believed security would be unable to control violent criminals or terrorists. Nearly half (41%) of directors also doubted that their security could handle an acute surge of patients and visitors greater than the ED and waiting room capacity; more than one-third (36%) reported that it was unlikely all points of hospital entry and egress could be secured within 15 minutes; and nearly two-thirds (62%) did not believe security protocols would be able to control traffic of incoming patients, additional hospital personnel, medical equipment suppliers, responding agencies, and media. Additionally, nearly one-third (31%) of respondents did not know of or have a security protocol to secure contaminated items, high-value possessions, or firearms, and 28% similarly did not know about the ability or use of a chain of custody for potential forensic evidence. These results highlight a huge vulnerability in homeland security and safety of hospital staff and patients. We recommend that all hospitals, regardless of size, develop protocols to ensure adequate resources for security in surge events and terrorist or imminent-threat events. These should be practiced routinely in drills and staff awareness of these plans should be promoted through easy access and frequent reinforcement.

Terrorist and mass casualty events are rare, and there have been multiple examples regarding how hospitals have been ill prepared to handle these surges. However, the well-run response after the Boston Marathon bombings in 2013 demonstrates a major event where protocols, coordinated efforts between different agencies, and disaster drills paid off.^12^ The Oso landslide that occurred outside a rural town in Washington in 2014 highlights that surge events can occur anywhere and all levels of hospitals must be prepared.

A national escalation of violent threats and acts against ED patients, visitors, and staff, coupled with increases in acute surge and mass casualty events, underscores the need to reevaluate and improve existing ED security capabilities. Results from this assessment highlight multiple shortcomings in ED security protocols and capabilities. These deficiencies are likely common outside of Washington as well and further research is needed to better describe the incidence of ED violence and security capabilities, ideally prospectively, in Washington and other states.

With the implementation of the Emergency Medical Treatment and Labor Act EMTALA in 1986, EDs must evaluate and stabilize all patients; however, they are not given adequate government resources for the protection of their staff. Smaller hospitals and communities may not have the resources to provide the same security measures that larger hospitals can afford, but these critical access hospitals are important resources themselves and their staff and patients are just as important to protect. Each community must create its own security and disaster plans and coordinate them with their local police forces. On a statewide basis, minimum security standards for daily operations and for mass security threats should be set and supported. Furthermore, it would be less costly and more efficient to create standard operating procedures for all hospitals within a state so that training could be more uniform, operations would be easier to coordinate during a disaster, and appropriate resource allocation could be ensured. Hospitals are a key resource for homeland security, and government financial resources should support protecting these facilities, staff, and patients.

## LIMITATIONS

Firstly, this was a survey instrument and we only collected data for a single state. This study relied on the knowledge, access to local records, and recollection of the Washington State ED directors without confirming response accuracy and with potential bias from the perception of local security resources and plans. Also, multiple interpretations of survey questions may have occurred and some questions were not answered by all respondents. For instance, we did not specify between types of mass disaster events. Finally, results have uncertain generalizability beyond Washington State. Due to anonymity we did not track dates of responses, and demographic data could not be analyzed alongside corresponding prevalence and response to threats or events.

## CONCLUSION

Our study reveals variable ED security staffing and training and a heterogeneous collection of plans and capabilities throughout Washington State. Although disaster plans exist, a number of common potential deficiencies are apparent, such as uniformity of security training, reporting frequency of violent acts, and specific protocols for securing firearms, high-value items, and forensic evidence. Concerning vulnerabilities exist including lack of readily available additional security, capability to rapidly secure access to EDs, and crowd and traffic control ability, and two-thirds of the ED directors we surveyed responded that resources were inadequate for day-to-day operations and surge events

ED security is increasingly critical given the progressive frequency of violent, acute surge, and mass casualty events. Although specific to Washington State, identified security deficiencies and vulnerabilities are likely shared and additional research should be considered by other EDs and regional emergency preparedness planners.

## Supplementary Information



## Figures and Tables

**Table 1:** Demographics of 78 EDs (emergency departments) that responded to a survey on security and preparedness for an acute surge or mass casualty event.

	n	%
Annual census	78	
<20,000	20	26
20,000–39,999	29	37
40,000–59,999	9	12
60,000–79,999	17	22
80,000–99,999	3	4
Practice environment	78	
Rural/critical access	24	31
Suburban 1–2k/mi^2^	11	14
Suburban 2–3k/mi^2^	19	24
Urban	24	31
Trauma level designation	78	
Level 1	1	1
Level 2	14	18
Level 3	23	29
Level 4	29	37
“Not Applicable”	11	14
# of Security personnel assigned to ED each shift	75	
“Not Applicable”	21	28
1	34	45
2	14	19
3	3	4
4	1	1
≥5	2	3
Timing of ED security coverage	75	
Never/not applicable	18	24
Special events	0	0
Daytime	4	5
Evenings	1	1
Nights/weekends	5	7
24-hour coverage	47	63
Source of security personnel	75	
“Not Applicable”	10	13
Hospital	45	60
Private/contracted company	13	17
Local law enforcement agency	7	9
Regional/state law enforcement agency	0	0
Training of security personnel assigned to ED	75	
“Not Applicable”	17	23
No formal or prior training	3	4
Prior security/law enforcement experience	11	15
By hospital/healthcare system	34	45
Agency or contractor sponsored course	8	11
Non-employer sponsored training course	2	3

**Table 2:** ED directors’ responses to a survey regarding response times and protocols during an acute surge or mass casualty event.

Question asked	Responded yes (%)	Responded no (%)
Could entry/egress from the hospital be secured in 15 minutes?	64%	36%
Could your security handle a violent criminal/terrorist?	57%	43%
Could your ED handle a surge of patients?	59%	41%
Could your ED handle a surge of patients’ family/friends?	56%	44%
In an acute surge of patients greater than the ED/waiting room capacity, does your ED have planned policies to limit visitors?	61%	39%
Does your ED have a protocol to control traffic for incoming patients, personnel, and supplies?	38%	62%

*ED,* emergency department
