# Supplementary material for: Security, Violent Events, and Anticipated Surge Capabilities of Emergency Departments in Washington State
Source: West J Emerg Med. 2017 Mar 3;18(3):466–73. doi: 10.5811/westjem.2016.10.30271 (PMC5391897; doi:10.5811/westjem.2016.10.30271)
Supplement: Supplementary file 1 [file wjem-18-466-s001.pdf]

Security in the Emergency Department

Emergency Department Directors of the State of Washington:

I am a second year Emergency Medicine resident at Madigan Army Medical Center located on Joint Base Lewis-McChord. I write to you with an opportunity to participate in a short, 15 minutes or less, anonymous survey pertaining to Emergency Department violence. The importance of this topic is undeniable in light of recent violent public events within our nation and abroad.

The purpose of this survey is to characterize the prevalence of violence across Washington's Emergency Departments, to obtain a broad understanding of security protocols, and the ability of these protocols to handle a "surge" or MASCAL events. This study hopefully represents the first of several potential studies about healthcare in WA State. The use of the ED Director List Serve is NOT expected to be frequent or common occurrence but a necessary one for this pilot study.

It is our plan to collect and analyze this data expeditiously in order to have the information available to all by March/April with the hopes that it will elucidate areas requiring further study and possibly help to further guide and employ security measures.

Participation is of course voluntary and anonymous but with 100% responses and with the best available information our data will be more robust and encompassing.

Thanks so much for your time and for your timely consideration in this matter.

Respectfully,

Jon Weyand, MD
CPT, MC
Emergency Medicine PGY-2

Demographics

*1. What is your annual census?

- < 20,000
- 20,000 - 39,999
- 40,000 - 59,999
- 60,000 - 79,000
- 80,000 - 99,999
- > 100,000

*2. What would best describe your current environment setting?

- Rural/Critical Access (pop. density less than 100 people/mi)
- Suburban - Lightly Populated (pop. density 1000-2000 people/mi)
- Suburban - Moderately Populated (pop. density 2000-3000 people/mi)
- Urban - (pop. density more than 3000 people/mi)

3. If urban, are you located in Seattle

- Yes
- No

*4. What is the trauma designation level for your hospital?

- Level I
- Level II
- Level III
- Level IV
- Not Applicable

Current ED Security

*5. On average, how many security personnel are present/assigned in your ED?

- ◯ Not Applicable
- ◯ 1
- ◯ 2
- ◯ 3
- ◯ 4
- ◯ >= 5

*6. If you have security personnel, how are they trained?

- ◯ Not Applicable
- ◯ No prior or formal training
- ◯ Prior security or law enforcement experience
- ◯ By hospital/health care system
- ◯ Agency or contractor sponsored course
- ◯ Non-Employer sponsored training course

*7. When are security personnel present/assigned in your ED?

- ◯ Never/Not Applicable
- ◯ Special Events
- ◯ Daytime
- ◯ Evenings
- ◯ Nights/Weekends
- ◯ 24-Hour Coverage

*8. Who provides your security personnel?

- ◯ Not Applicable
- ◯ Hospital
- ◯ Private/Contracted Company
- ◯ Local Law Enforcement Agency
- ◯ Regional/State Law Enforcement Agency

Security in the Emergency Department

***9. If needed, does a plan exist to notify and receive additional security personnel within the hospital?**

- ◯ Yes
- ◯ No

***10. How long would it take to notify and receive additional security personnel within the hospital?**

- ◯ Not applicable
- ◯ < 5 minutes
- ◯ 5-15 minutes
- ◯ 16-30 minutes
- ◯ 31-60 minutes
- ◯ > 60 minutes

***11. In the general consensus of ED staff/faculty, is the current level of security in your ED adequate?**

- ◯ Yes
- ◯ No

General Planning

***12. Is the availability and size of additional security response to your ED during a disaster/mass casualty event adequate (time to arrival of first security personnel)?**

- ◯ Yes
- ◯ No
- ◯ Do Not Know

***13. Have you witnessed or heard about a violent threat or act towards:**

	Yes	No
Admin personnel (ie Clerk)	◯	◯
Nursing staff	◯	◯
Provider	◯	◯
Security personnel	◯	◯
Another patient	◯	◯
Family or friends of a patient	◯	◯

***14. If the answer above was yes, was the event reported to your hospital's administration?**

	Yes	No
Admin Personnel (i.e. Clerk)	◯	◯
Nursing staff	◯	◯
Provider	◯	◯
Security personnel	◯	◯
Another patient	◯	◯
Family or friends of a patient	◯	◯

***15. If your hospital's Emergency Management Plan was activated, how many additional security personnel within the hospital would be available in your ED within 15 minutes?**

- ◯ None
- ◯ 1
- ◯ 2
- ◯ 3
- ◯ 4
- ◯ >=5

Security in the Emergency Department

16. If your hospital's Emergency Management Plan was activated, how many additional security personnel within the hospital would be available in your ED within 30 minutes?

- ◯ None
- ◯ 1
- ◯ 2
- ◯ 3
- ◯ 4
- ◯ >=5

Scenario Based Planning:

If the following disaster/mass casualty event occured near your hospital, do you and your staff believe that the security personnel in your ED would be able to control the following scenarios?

***17. Who would be or supply the first additional responding security personnel?**

- ◯ Not applicable
- ◯ Hospital
- ◯ Private/Contracted Company
- ◯ Local law enforcement agency
- ◯ Regional/State law enforcement agency

18. Does your facility's Emergency Management Plan contain a security plan in the event of a disaster/mass casualty event?

- ◯ Yes
- ◯ No

***19. What is the highest level of assurance you have that additional security personnel would respond immediately?**

- ◯ Not Applicable
- ◯ Not Sure/Assumption/Common Knowledge
- ◯ Already present in the facility/campus
- ◯ Unwritten Agreement
- ◯ Contract
- ◯ Past/Recent Memorandum of Understanding/Agreement (> 24 months ago)
- ◯ Current Memorandum of Understanding/Agreement (< 24 months ago)

20. Secure all points of entry to/egress from your hospital in 15 minutes?

- ◯ Yes
- ◯ No

***21. Violent criminal(s) or terrorist(s) present in the ED?**

- ◯ Yes
- ◯ No

Security in the Emergency Department

***22. Surge of incoming patient with an hour (>= than current capacity of your ED waiting room and department)?**

- ◯ Yes
- ◯ No

***23. Surge of of incoming family members and friends of patients within an hour?**

- ◯ Yes
- ◯ No

***24. If a surge of a patients greater than the current capacity of your ED waiting room and department occurs, do you have a planned policy to limit access of patient visitors to the ED?**

- ◯ Yes
- ◯ No

25. Traffic control for incoming patients, supplies (i.e. blood products, medications, medical equipment, etc.), media, responding agencies, and incoming hospital personnel within 15 minutes?

- ◯ Yes
- ◯ No

26. Quarantine contaminated or contagious patients?

- ◯ Yes
- ◯ No

27. Secure contaminated, high value item or firearms?

- ◯ Yes
- ◯ No

28. Secure and maintain chain of custody of potential forensic evidence?

- ◯ Yes
- ◯ No
